# Supplementary material for: Structural bioinformatics studies of six human ABC transporters and their AlphaFold2-predicted water-soluble QTY variants
Source: QRB Discov. 2024 Jan 19;5:e1. doi: 10.1017/qrd.2024.2 (PMC10988169; doi:10.1017/qrd.2024.2)
Supplement: Pan et al. supplementary material [file S2633289224000024sup001.docx]

**Supplementary Materials**

**Structural bioinformatics studies of six human ABC transporters and their AlphaFold2 predicted water-soluble QTY variants**

Emily Pan^1^, Fei Tao^2^, Eva Smorodina^3^, and Shuguang Zhang^4,*^

**Figure S1. The enlarged alignments of 6 human ABC transporters a) ABCB7, b) ABCC8, c) ABCD1, d) ABCD4, e) ABCG1 and f) ABCG5** **between native protein and their QTY variants.**


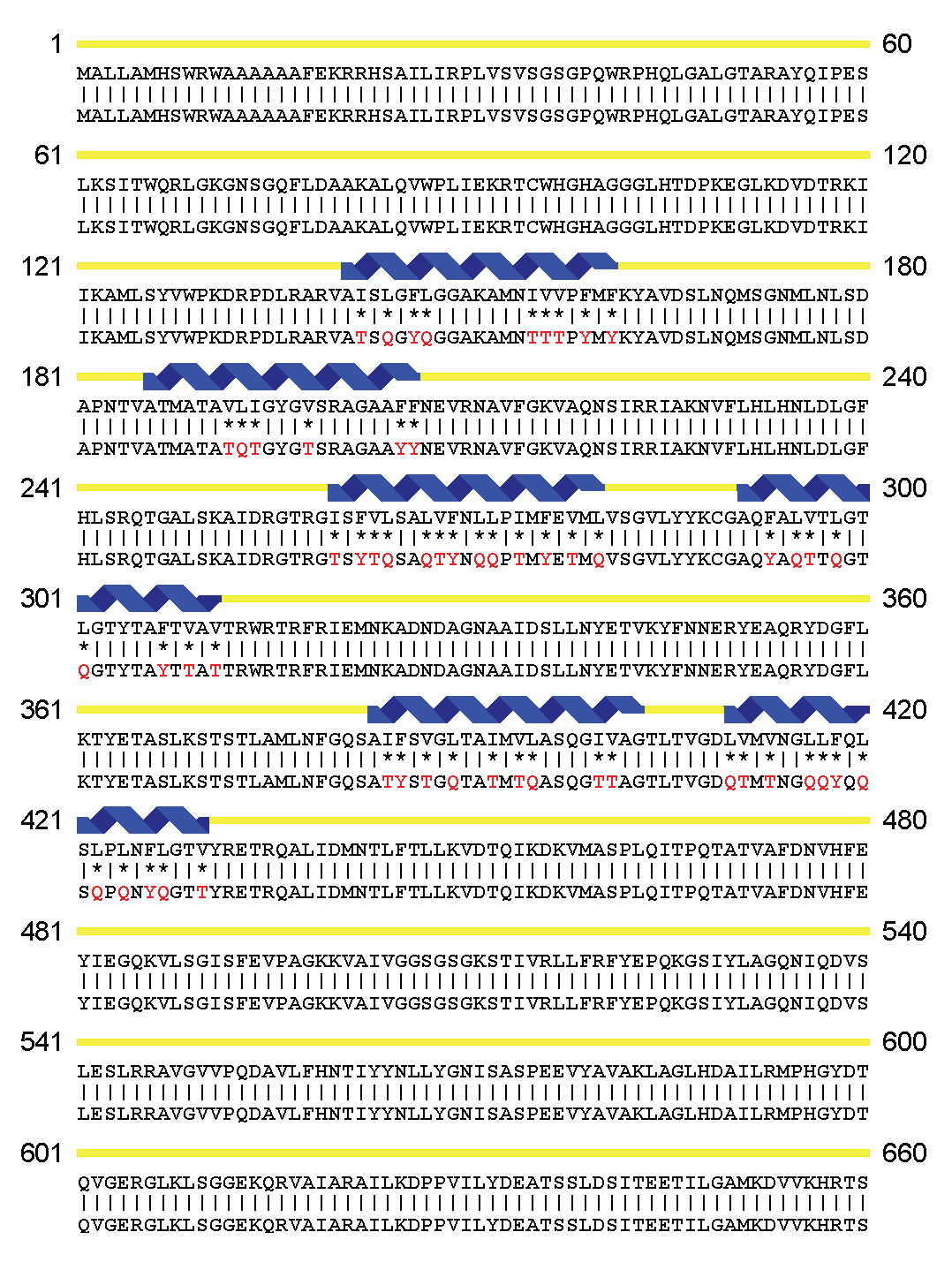


**Figure S1. a, ABCB7 vs ABCB7^QTY^.** The symbols | and * indicate whether amino acids are identical or different, respectively. Please note the Q, T, and Y amino acids (red) replacing L, V and I, and F, respectively. The alpha-helices (blue) are shown above the protein sequences. The loop color codes are: internal (yellow) and external (red).


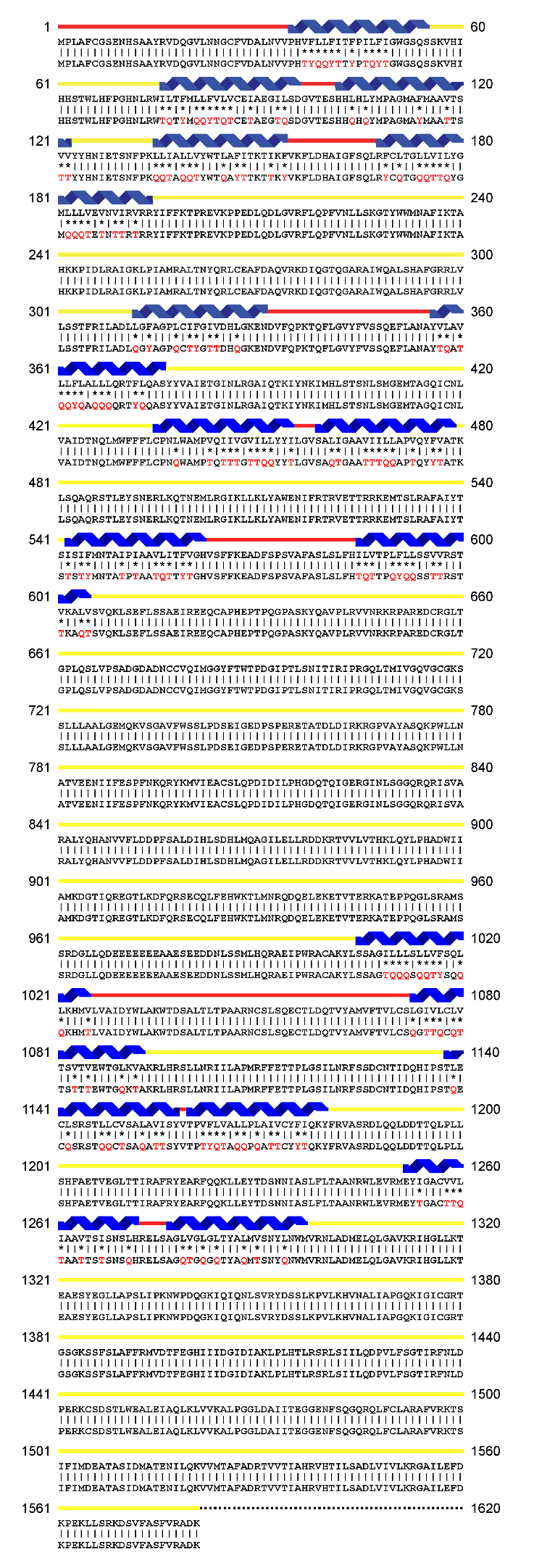


**Figure S1. b, ABCB8 vs ABCB8^QTY^.** The symbols | and * indicate whether amino acids are identical or different, respectively. Please note the Q, T, and Y amino acids (red) replacing L, V and I, and F, respectively. The alpha-helices (blue) are shown above the protein sequences. The loop color codes are: internal (yellow) and external (red).


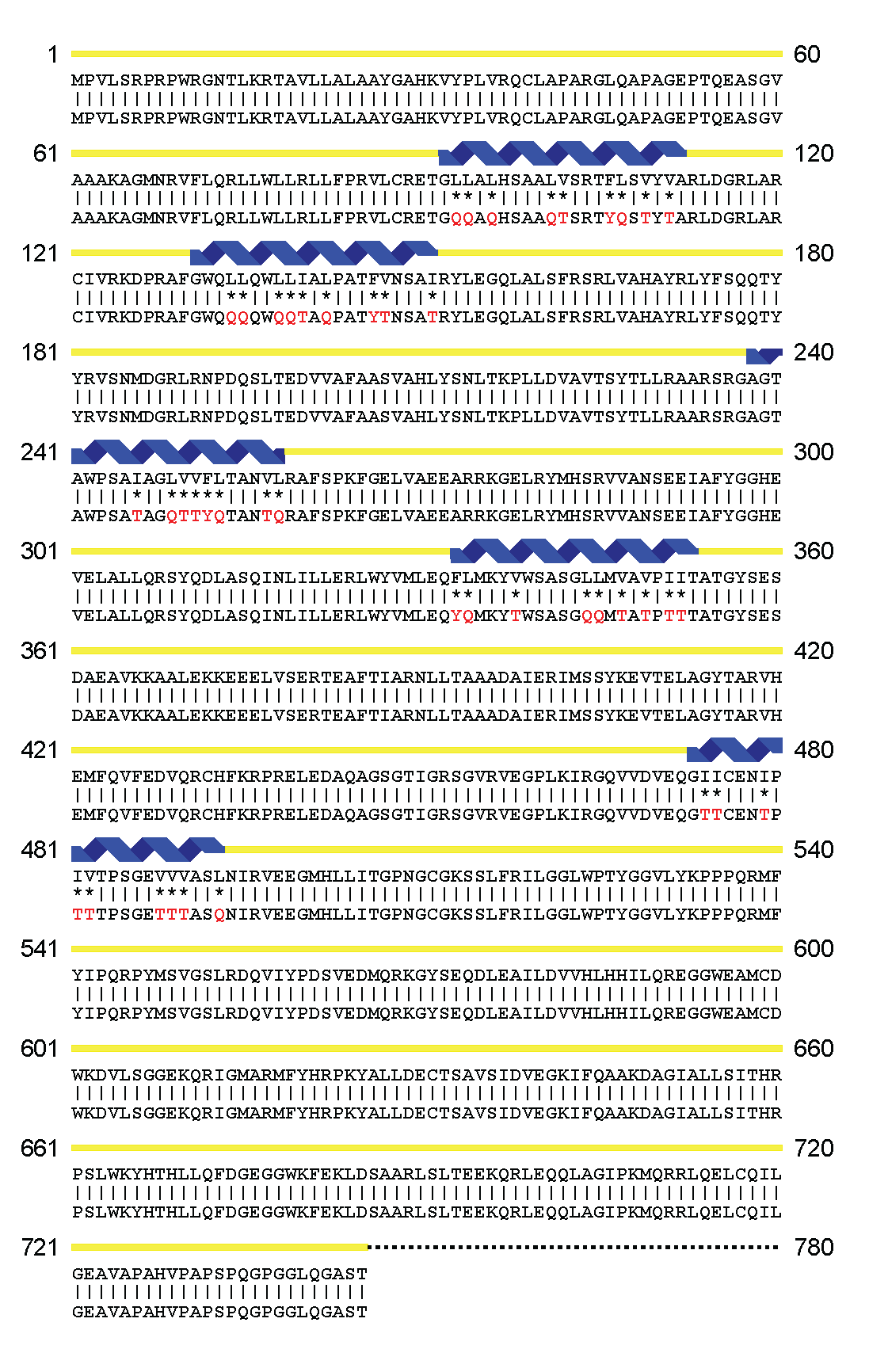


**Figure S1. c, ABCD1 vs ABCD1^QTY^.** The symbols | and * indicate whether amino acids are identical or different, respectively. Please note the Q, T, and Y amino acids (red) replacing L, V and I, and F, respectively. The alpha-helices (blue) are shown above the protein sequences. The loop color codes are: internal (yellow) and external (red).


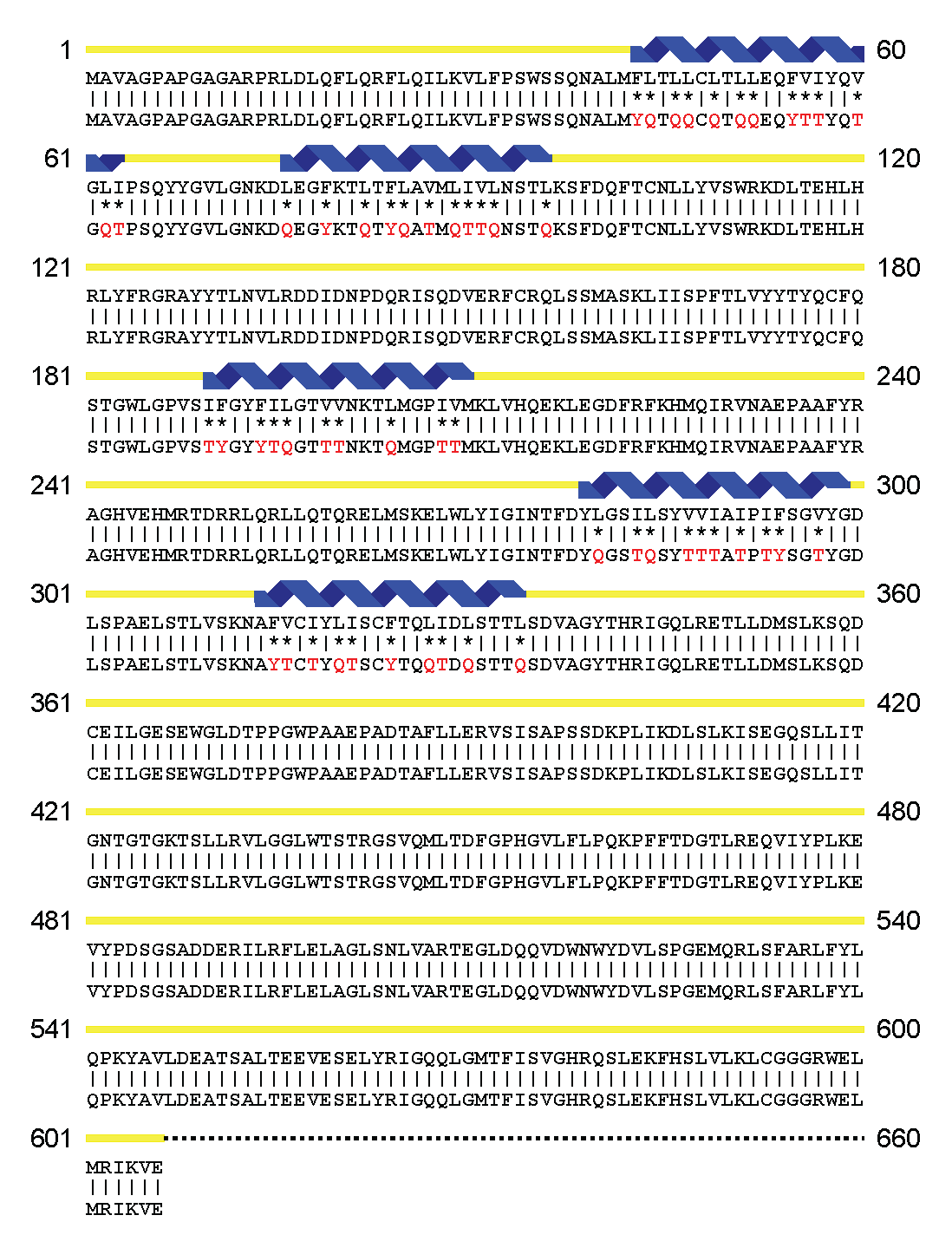


**Figure S1. d, ABCD4 vs ABCD4^QTY^.** The symbols | and * indicate whether amino acids are identical or different, respectively. Please note the Q, T, and Y amino acids (red) replacing L, V and I, and F, respectively. The alpha-helices (blue) are shown above the protein sequences. The loop color codes are: internal (yellow) and external (red).


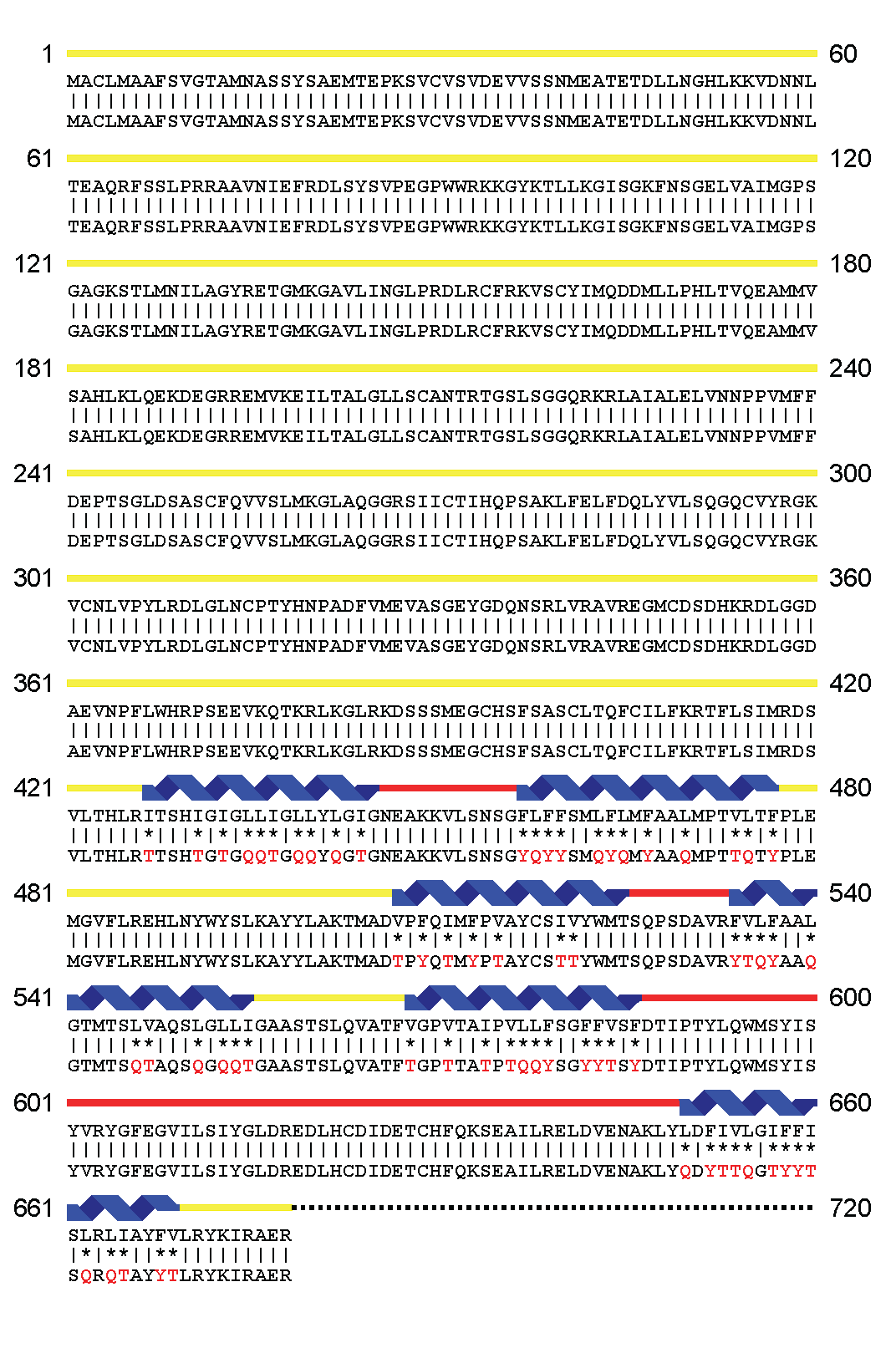


**Figure S1. e, ABCG1 vs ABCG1^QTY^.** The symbols | and * indicate whether amino acids are identical or different, respectively. Please note the Q, T, and Y amino acids (red) replacing L, V and I, and F, respectively. The alpha-helices (blue) are shown above the protein sequences. The loop color codes are: internal (yellow) and external (red).


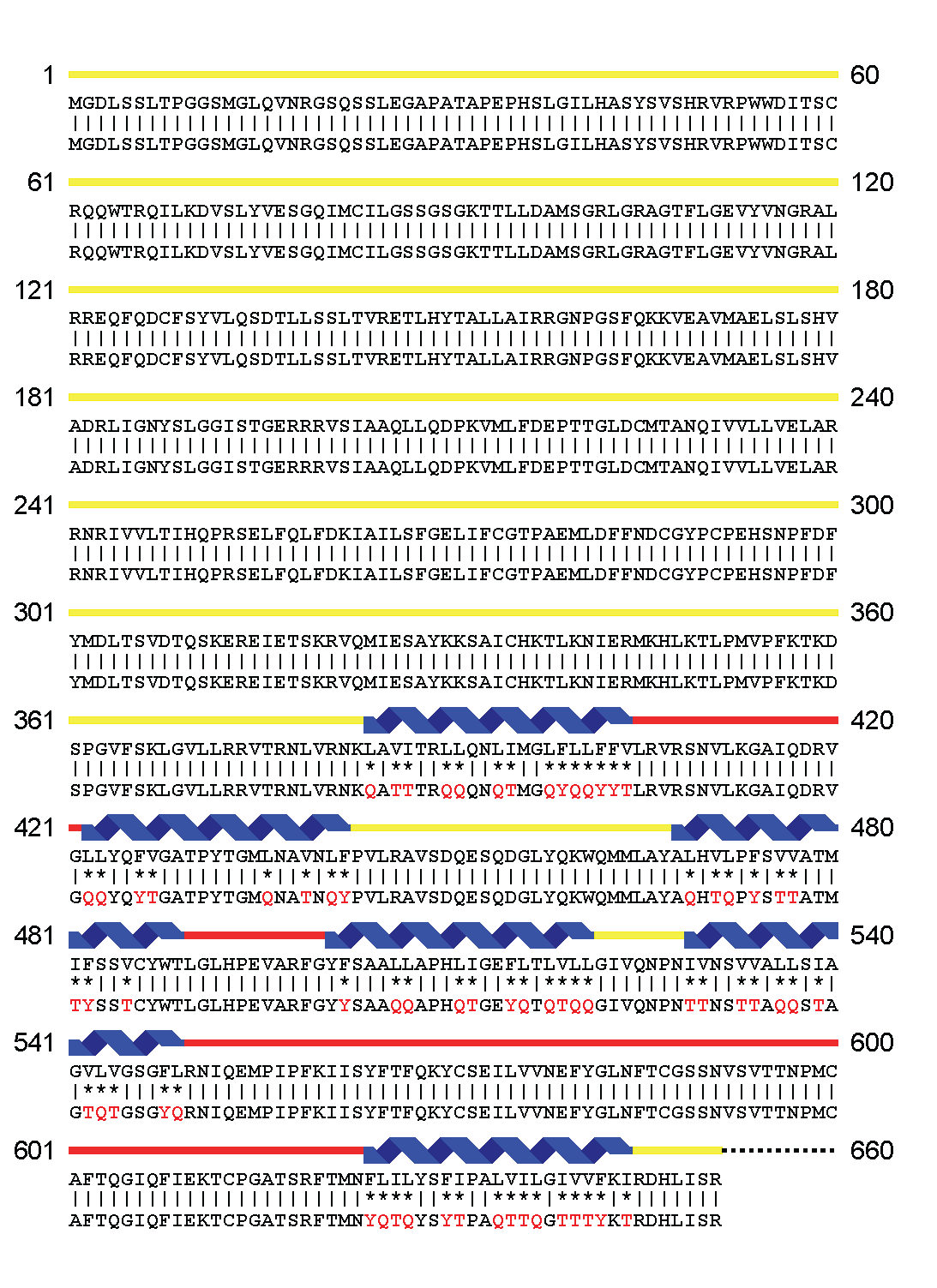


**Figure S1. f, ABCG5 vs ABCG5^QTY^.** The symbols | and * indicate whether amino acids are identical or different, respectively. Please note the Q, T, and Y amino acids (red) replacing L, V and I, and F, respectively. The alpha-helices (blue) are shown above the protein sequences. The loop color codes are: internal (yellow) and external (red).

**
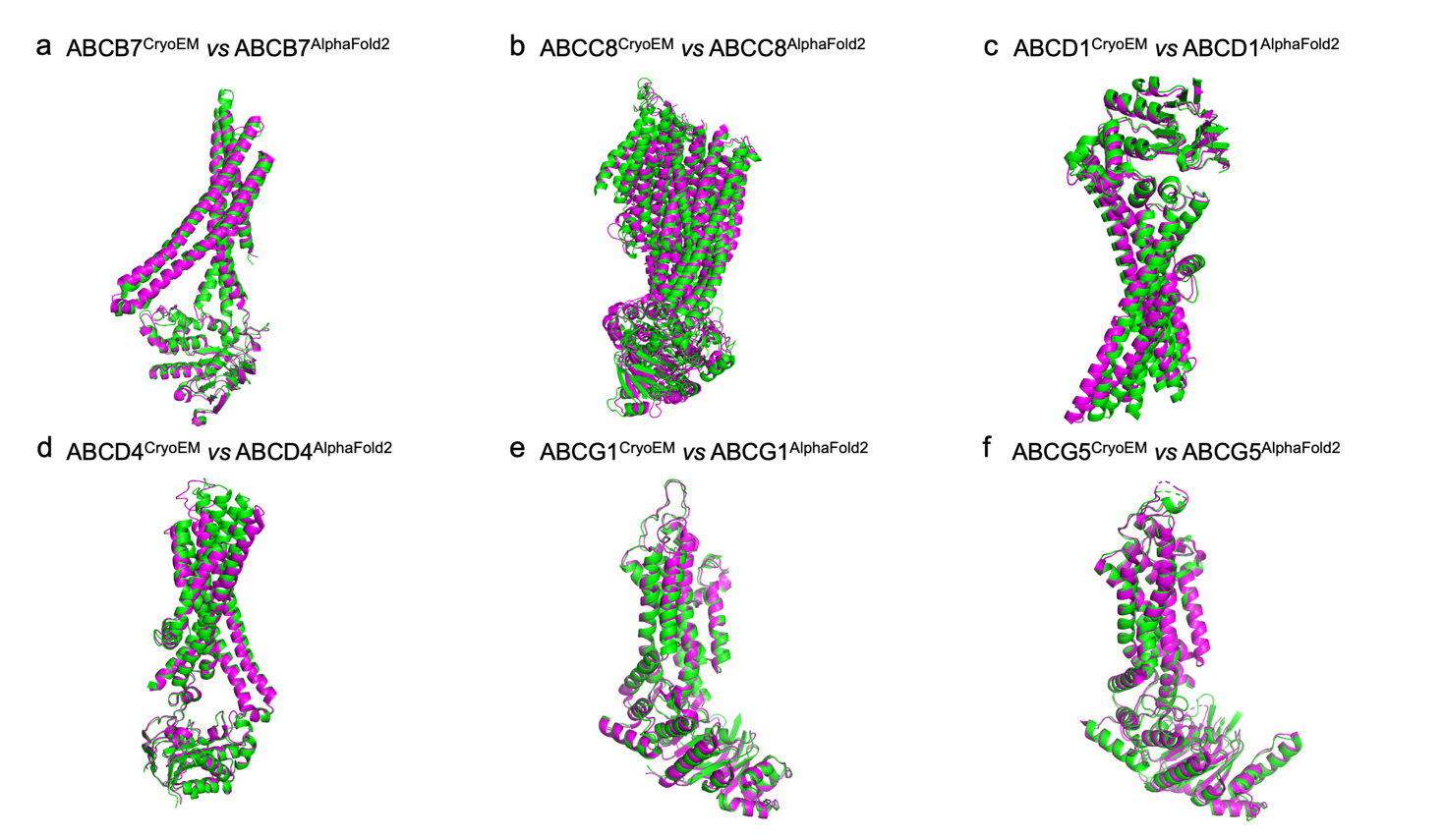
**

**Figure S2. Superpositions of CryoEM structures and the AlphaFold2 predicted native structures.** Color code: magenta= CryoEM structures, green= native AF2 predicted structures.  **a)** ABCB7^CryoEM^ *vs* ABCB7^AF2-native^ (RMSD=1.649Å), **b)** ABCC8^CryoEM^ *vs* ABCC8^AF2-native^ (RMSD=3.411Å), **c)** ABCD1^CryoEM^ *vs* ABCD1^AF2-native^ (RMSD=2.235Å), **d)** ABCD4^CryoEM^ vs ABCD4^AF2-native^ (RMSD=1.070Å), **e)** ABCG1^CryoEM^ *vs* ABCG1^AF2-native^ (RMSD=1.262Å) and **f)** ABCG5^CryoEM^ *vs* ABCG5^AF2-native^ (RMSD=0.896Å).

**Superposed transmembrane alpha-helices of the ABC transporters only**

We superimposed the transmembrane domains by cutting out everything that is not a part of the transmembrane domains. Although some of the ABC transporters including ABCB7, ABCC8, ABCG1 and ABCG5/G8 superposed very well with their QTY variants having RMSD ~1Å or <1Å; on the other hand, ABCD1 and ABCD4 have RMSD 2.338Å and 3.787Å, respectively. The transmembrane domains are not adjusted after amino acids in the non-transmembrane domain are removed. Here are the superposed CryoEM structures and their AlphaFold2 predicted water-soluble QTY variants with RMSD values.


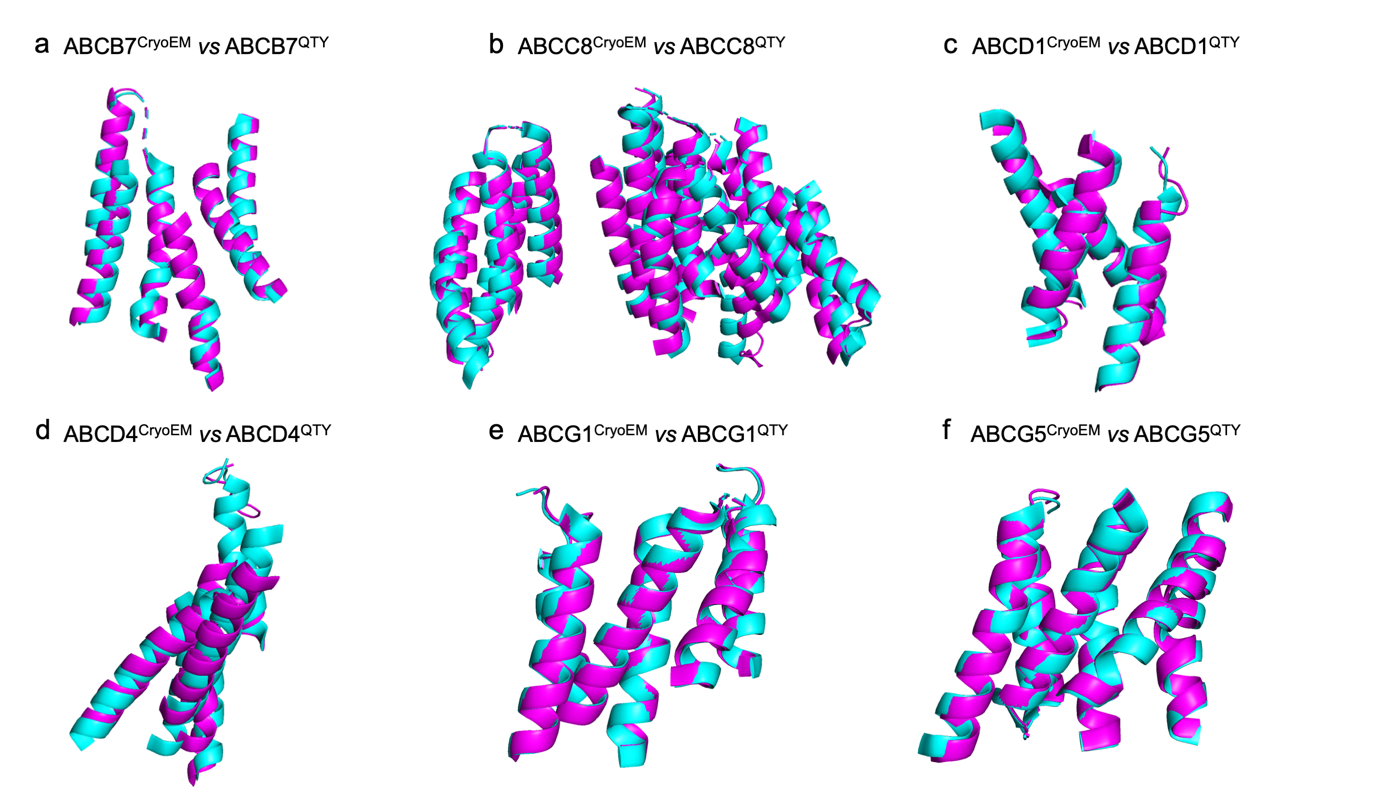


**Figure S3. Superposed transmembrane alpha-helices of the ABC transporters only. a**) ABCB7^CryoEM^ *vs* ABCB7^QTY^ (RMSD=0.686Å), **b**) ABCC8^CryoEM^ *vs* ABCC8^QTY^ (RMSD=1.390Å), **c**) ABCD1^CryoEM^ *vs* ABCD1^QTY^ (RMSD=2.338Å), **d**) ABCD4^CryoEM^ *vs* ABCD4^QTY^ (RMSD=3.787Å), **e**) ABCG1^CryoEM^ *vs* ABCG1^QTY^ (RMSD=0.539Å, **f**) ABCG5/G8^CryoEM^ *vs* ABCG5/G8^QTY^ (RMSD=0.701Å).
